# Lessons From the Field From a Volunteer Telehealth Ambassador Program to Enhance Video Visits Among Low-Income Patients: Qualitative Improvement Study

**DOI:** 10.2196/49993

**Published:** 2024-04-15

**Authors:** Delphine S Tuot, Aarya Mukherjee, Amanda Churape, Triveni DeFries, George Su, Elaine C Khoong, Courtney Lyles

**Affiliations:** 1 Department of Medicine Zuckerberg San Francisco General Hospital University of California, San Francisco San Francisco, CA United States; 2 Center for Innovation in Access and Quality at Zuckerberg San Francisco General Hospital University of California, San Francisco San Francisco, CA United States; 3 UCSF Center for Vulnerable Populations Zuckerberg San Francisco General Hospital San Francsico, CA United States; 4 University of California, Berkeley Berkeley, CA United States; 5 Department of Epidemiology and Biostatistics University of California, San Francisco San Francisco, CA United States; 6 Center for Healthcare Policy and Research University of California, Davis Sacramento, CA United States; 7 Department of Public Health Sciences University of California Davis School of Medicine Sacramento, CA United States

**Keywords:** digital barriers, digital support, digital technologies, equity, health care delivery, safety-net, telehealth

## Abstract

**Background:**

The prevalence of telehealth video use across the United States is uneven, with low uptake in safety-net health care delivery systems, which care for patient populations who face barriers to using digital technologies.

**Objective:**

This study aimed to increase video visit use in an urban safety-net delivery system. We piloted a telehealth ambassador program, in which volunteers offered technical support to patients with access to digital technologies to convert primary care visits already scheduled as telehealth audio-only visits to telehealth video visits.

**Methods:**

We used a descriptive approach to assess the feasibility, efficacy, and acceptability of the pilot telehealth ambassador program. Feasibility was quantified by the percentage of eligible patients who answered calls from telehealth ambassadors. Program efficacy was measured in two ways: (1) the percentage of patients with access to digital technology who interacted with the navigators and were successfully prepared for a telehealth video visit, and (2) the percentage of prepared patients who completed their scheduled video visits. Program acceptability was ascertained by a structured telephone survey.

**Results:**

Telehealth ambassadors attempted to contact 776 eligible patients; 43.6% (338/776) were reached by phone, among whom 44.4% (150/338) were provided digital support between March and May 2021. The mean call duration was 8.8 (range 0-35) minutes. Overall, 67.3% (101/150) of patients who received support successfully completed a telehealth video visit with their provider. Among the 188 patients who were contacted but declined video visit digital support, 61% (114/188) provided a reason for their decline; 42% (48/114) did not see added value beyond a telehealth audio-only visit, 20% (23/114) had insufficient internet access, and 27% (31/114) declined learning about a new technology. The acceptability of the telehealth ambassador program was generally favorable, although some patients preferred having in-real-time technology support on the day of their telehealth video visit.

**Conclusions:**

This high-touch program reached approximately one-half of eligible patients and helped two-thirds of interested patients with basic video visit capability successfully complete a video visit. Increasing the program’s reach will require outreach solutions that do not rely solely on phone calls. Routinely highlighting the benefits of video visits, partnering with community-based organizations to overcome structural barriers to telehealth use, and offering in-real-time technology support will help increase the program’s efficacy.

## Introduction

The onset of the COVID-19 pandemic in March 2020 led to an unprecedented era of digital reliance resulting from quarantining and shelter-in-place precautions. The dependence on digital interactions as opposed to in-person communication disrupted health care delivery with the rapid rollout of telehealth services across the United States, including telehealth audio-only visits (ie, telephone visits) and telehealth video visits [1]. The prevalence of telehealth video visit use across different care delivery settings was uneven, however, with particularly low uptake in safety-net systems, which often consist of county hospitals, health clinics, and emergency departments that treat patients regardless of their ability to pay or their immigration status [2,3]. Reasons for low telehealth video uptake in safety-net systems are multifactorial, including suboptimal infrastructure for implementation as well as challenges faced by their patient populations due to structural and socioeconomic barriers and limited digital literacy [4,5]. There is a paucity of trial data that directly compares different telehealth visit modalities, and both likely have a role to play in primary care delivery [6]. However, retrospective studies examining data from electronic health records suggest that primary care telehealth video visits are associated with more clinical actions, including more medication prescriptions and diagnostic testing and fewer return in-person visits within 7 days compared to telehealth audio-only visits [7]. Additionally, telehealth video visits have been associated with less clinician concern for patient safety compared with telehealth audio [8].

Patient satisfaction (overall and within safety net settings) with telehealth services, including, but not limited to, telehealth video, has been generally favorable [9]. However, many patient groups have concomitantly voiced hesitation surrounding the shift from in-person to remote care delivery, including the use of patient portals and telehealth video visits [10]. These groups include individuals with low digital literacy, defined by a poor ability to use information technologies to find, evaluate, create, and communicate information, as well as those with low socioeconomic status. The presence of these conflicting messages is reinforced by data that suggest that socioeconomic disparities in health technology use are not primarily driven by low patient interest but rather by suboptimal knowledge or digital skills or both [11]. There is a need for robust training and support to bridge the gap between interest in telehealth and the actual use of digital technology.

During the early phase of the pandemic, the use of telehealth video visits in our urban safety-net health care delivery system required patients to download video platform software on their computer or mobile device. Efforts to support patients through this process revealed the need for substantial staff time to provide one-on-one, tailored technical support to match patients’ language, literacy, digital literacy level, and technical needs. Other safety-net health delivery systems had similar experiences, reinforcing the need for individualized counseling on how to use telehealth video services [12]. Our system focused its efforts on addressing one barrier in increasing uptake of telehealth video visits in our low-income population—that of low confidence in using digital tools to participate in telehealth video visits. Here, we describe the feasibility, efficacy, and acceptability of one pilot initiative—a telehealth ambassador program—that offered technical support to patients with access to digital technologies to convert primary care visits already scheduled as telehealth audio-only visits to telehealth video visits.

## Methods

### Study Setting and Patient Population

San Francisco Health Network (SFHN) is the integrated public health care delivery system that serves San Francisco’s low-income population. It consists of the Zuckerberg San Francisco General Hospital; a long-term care facility; and full-spectrum ambulatory care services delivered through 14 primary care clinics, jail health, specialty care clinics, and a variety of community-based programs. Its primary care clinics, in which this pilot program was conducted, serve 59,000 individual patients with 310,270 annual encounters. Patients are racially and ethnically diverse: 37% Latinx, 20% Asian, 18% White, 15% Black or African American, and 10% other or unknown, and one-third have limited English proficiency. Consistent with its safety-net health system designation, nearly 100% of SFHN patients are covered by government-sponsored insurance: 58% Medicaid, 32% Medicare, and 9% to 10% San Francisco County health access plans.

### Telehealth Ambassador Program

Second-year preclinical medical students were given the opportunity to serve as volunteer telehealth ambassadors as part of a quality improvement course embedded in their medical school curriculum. Leveraging role-playing activities, students were trained by the quality improvement team to call patients, introduce themselves, offer technical support, and troubleshoot potential barriers to completing a telehealth video visit. Over the subsequent 10 weeks in early 2021, the telehealth ambassadors reached out to patients with telehealth audio-only appointments scheduled with a small number of primary care providers using a standardized script to introduce themselves as extensions of the primary care clinical team and confirm patients’ identities and upcoming scheduled telehealth audio-only appointments. Each eligible patient received at least 2 phone calls. The telehealth ambassadors then screened patients with standardized questions gauging: (1) willingness to participate in video visits; (2) access to a digital device; and (3) access to sufficient data for telehealth video visits, identified by asking about the use of web-based communication platforms (ie, Facebook, Facetime, or WhatsApp). In the absence of a gold standard screening tool for video visit use, the inclusion of these screening questions has been recommended as a best practice for enhancing patient engagement with digital technologies [13]. Among patients with access to digital technology and sufficient data to participate in a telehealth video visit, telehealth ambassadors helped patients download the videoconference app used by our health system on their internet-enabled device and offered a practice session to confirm their ability to use the software. Successful “on-boarding” of an individual patient included a complete download of the video visit app with audio and visual checks and participation in a practice session with the telehealth ambassador. Professional telephone interpreters were available to help patients with limited English proficiency. After successful onboarding, a member of the team with access to the electronic medical record then converted the patient’s upcoming appointment from a telehealth audio visit to a telehealth video appointment.

### Ethical Considerations

This was a quality improvement project aimed at assessing the feasibility, efficacy, and acceptability of a new navigator program meant to increase the delivery system’s use of telehealth video. The quality improvement team did not collect or store patient-level, identifiable data. Telehealth ambassadors did not have access to patient electronic health records; they only had access to patient name, telephone number, and primary care clinician information. Such quality improvement activities do not require institutional review board approval at our institution (University of California, San Francisco), even if they include patient surveys, even if they include patient surveys, as long as the primary purpose of the survey is to gauge the opinions and perceptions of customers to improve the delivery of health care. As such, patients did not participate in a formal consent process to interact with the telehealth ambassadors, although they had the opportunity to decline telehealth ambassador services. Patients contacted after their scheduled video visit could also decline answering questions about their experience.

### Analysis

We used a descriptive approach to assess the feasibility, efficacy, and acceptability of the pilot telehealth ambassador program. Feasibility was quantified by the percentage of eligible patients who answered calls from telehealth ambassadors. The efficacy of the program was measured in two ways: (1) the percentage of patients willing to participate in a telehealth video visit with access to digital technology who interacted with the navigators and were successfully “onboarded” and (2) the percentage of onboarded patients who completed their scheduled video visits. Telehealth ambassadors called patients within 2 weeks after their scheduled video visit, using a brief, standardized set of survey prompts (Textbox 1) that were developed by the team with input from operational leaders to ascertain the acceptability of the telehealth ambassador program. Feedback about the telehealth ambassador program was reviewed by the 2 members of the quality improvement team (DST and AC). Barriers to video visit completion were tabulated using descriptive statistics.

**Acceptability survey prompts.**

1. Did you successfully complete the video visit (yes or no)?
2. Before having the setup call with a Telehealth Ambassador, how successful did you think you would be with completing the video visit (not at all, a little, somewhat, or very)?
3. Would you recommend a Telehealth Ambassador phone call to other patients to get set up for a video visit?
4. What would help you feel prepared to make the most out of a video visit?
5. What type of visit would you prefer for your next visit?

## Results

All patients who had scheduled telehealth audio-only visits with a few primary care providers were eligible to be contacted by a telehealth ambassador (n=776). While we did not collect sociodemographic data for those 776 patients, SFHN primary care patients are racially, ethnically, and linguistically diverse (Table 1). During the study period, primary care patients with telehealth visits (n=12,851) compared to those with in-person visits (n=8345) were more likely to be aged between 45 and 64 years, female, of Asian race and non-Hispanic ethnicity, and speak a language other than English (Table 1).

**Figure 1:**
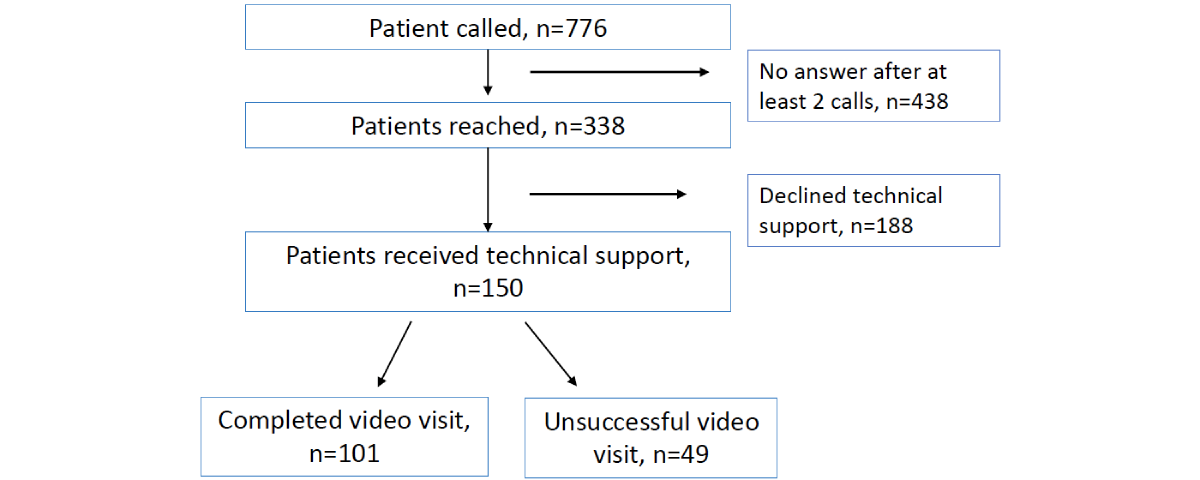
Consolidated Standards of Reporting Trials (CONSORT) diagram of participants.

Telehealth ambassadors attempted to contact all 776 eligible patients with future telehealth audio visits scheduled with a participating primary care provider (Figure 1). Overall, 43.5% (338/776) of patients were reached by phone. Of those, 44.4% (150/338) voiced interest in participating, had access to digital technology and data, and were successfully onboarded after receiving ambassador support, which included a download of the video visit app and confirmation that the patient could use the software, most often with a practice video call. The mean call duration between telehealth ambassadors and the 150 patients was 8.75 (range 0-35) minutes.

Nearly two-thirds (101/150; 67.3%) of patients who were interested, received support, and were deemed ready for a video visit successfully completed their scheduled telehealth video visit with their provider. This translated into a 30% (101/338) video visit completion rate among eligible patients reached by phone. Brief phone surveys with patients who did not have a successful video visit revealed a variety of factors that impaired the video visit completion rate: patient nonattendance to their telehealth visit; internet or connection challenges on the day of the visit, which led to the provider recommending a switch to an audio-only visit; and a last-minute patient preference for a phone visit.

**Table 1:** Sociodemographic characteristics of San Francisco Health Network patients with primary care visits between January and February 2021.

Characteristics			In-person (n=8345), n (%)		Telehealth (n=12,851), n (%)		*P* value	
**Age range (years)**								<.001
	≤17	1528 (18.3)		1021 (8.9)				
	18-44	1856 (22.2)		2878 (22.4)				
	45-64	3015 (36.1)		5319 (41.4)				
	≥65	1946 (23.3)		3633 (28.3)				
**Sex**								.002
	Female	4551 (54.5)		7318 (56.9)				
	Male	3793 (45.5)		5530 (43)				
	Other	1 (0.01)		3 (0.02)				
**Race**								<.001
	White	1120 (13.4)		2002 (15.6)				
	Black or African American	1434 (17.2)		1827 (14.2)				
	Asian	1520 (18.2)		3625 (28.2)				
	Other	4213 (50.5)		5298 (41.2)				
	Unknown	58 (0.7)		100 (0.7)				
**Ethnicity**								<.001
	Hispanic	3747 (44.9)		4681 (36.4)				
	Non-Hispanic	4540 (54.4)		8060 (62.7)				
	Unreported	58 (0.7)		100 (0.7)				
**Primary language**								.006
	English	4189 (50.2)		6164 (48)				
	Other	4152 (49.8)		6679 (52)				
	Unreported	4 (0.05)		8 (0.06)				

A total of 55.6% (188/338) of individuals reached by telehealth ambassadors declined technical support and thus declined changing their audio-only visit to a telehealth video visit. A total of 60.6% (114/188) provided a reason for their decline. In all, 42.1% (48/114) believed video visits did not add value above a telehealth audio visit, 20.2% (23/114) had insufficient internet access or did not have an internet-enabled device, 27.2% (31/114) were hesitant to learn new technologies, 4.4% (5/114) reported time constraints limiting their ability to learn how to download the videoconferencing app, and 6.1% (7/114) did not have a safe or private space to conduct video visits.

Nearly one-third (45/150, 30%) of individuals who received technical support responded to the brief acceptability survey. Most patients appreciated the telehealth ambassador phone call and preparatory support and recommended that the program continue. This was particularly true among those who successfully completed their video visits. Example quotes include “I would recommend it absolutely; the set-up call was very helpful” and “I would recommend a set-up call to others who are not familiar with using (the telehealth video software).” However, some patients still lacked confidence in participating in a video visit on the day of their appointment and relied on family members to successfully complete the video visit. A patient mentioned: “I still need help from family members [on the day of the visit]. [With the volunteer], it was set up successfully, but it didn’t work the day of the appointment.” Reactions were more mixed among patients who were not successful at completing their video visits despite receiving technical support. Some patients regarded the program favorably: “I was texted a link for the video visit, but I was not able to connect...The set-up call would likely help others have a successful video visit.” Others felt that the technical support was insufficient, particularly if they were alone, and stated a preference for technical support on the day of appointment: “It was difficult by myself. An IT call right before would be the best solution.”

## Discussion

### Overview

The SFHN telehealth ambassador volunteer program offered one-on-one remote technical support to patients to increase access to video visits during the COVID-19 pandemic. Overall, this high-touch program reached approximately one-half of eligible patients and helped two-thirds of interested patients with basic video visit capability successfully complete a video visit. Data offered insights into how we could revise the program to achieve greater success, which we define as an increase in the percentage of telehealth appointments that are video versus audio only among individuals equipped with the digital tools that allow them to participate in video visits.

The SFHN telehealth ambassador program relied on telephone communication to reach patients. Failure to make this initial connection represented a missed opportunity to offer digital support to patients. Increasing patient awareness of video visit availability through marketing campaigns and offering a centralized telehealth support desk for patients and families to call for support rather than waiting for a telehealth ambassador phone call could facilitate participation from a higher number of patients [14,15]. Automating the initial phone call or leveraging SMS text messaging might also help. Previous research has shown the practicality of automated interactive voice response apps to make large volumes of calls for patient outreach [16]. Importantly, these calls or texts would need to be multilingual and easily transferred to a telehealth ambassador with professional telephone interpreter support to successfully onboard patients and families who answered the automated phone call or reached out for additional help after receiving a SMS text message. These changes would increase the overall reach of the program, though they would not likely enhance efficacy.

Among those individuals who declined video visit support, many did not feel as though video visits added value to their health care compared to audio-only visits. This is consistent with data from the 2019-2020 nationally representative Medicare Current Beneficiary Survey, which suggested that 28.5% of patients with video visit experience prefer an audio-only visit over a telehealth video visit when clinically appropriate. Ambivalence about video visits has been overcome when routinely offered by health care teams, especially when they highlight provider preference for video visits over audio-only visits [17]. Similarly, frequent and repeated recommendations and offers to help patients use web-based portals have been associated with higher patient portal engagement [18,19]. Incorporating the telehealth ambassador program into clinical workflows will thus be key to increasing interest in video visits among eligible patients. In our next iteration, we are asking all primary care clinic personnel, including clinicians, nurses, medical assistants, front desk staff, nutritionists, etc to routinely recommend video visits over audio-only visits, even to patients who have previously declined a video visit, and simultaneously to refer patients to the telehealth ambassador team for digital support.

We are also recommending that telehealth ambassadors strongly encourage patients to have a telehealth video partner at home who can help them connect on the day of the visit. This stems from patient feedback among those who were and were not successful at completing their video visits. Such a partner could include a family member, or a community member, or a volunteer, from a community-based organization.

Approximately 20% of patients reached by phone reported not having access to an internet-capable device or an unlimited data plan to facilitate video visits. While data from the San Francisco Department of Technology suggests that 87% of the San Francisco general population has high-speed internet access, this percentage is far lower among patients from racial or ethnic minority backgrounds and residents who are low-income, older, have limited English proficiency, or have a disability [20]. Empowering telehealth ambassadors to refer these individuals to partnering community-based organizations that can help them access low-cost devices will be a key feature of our next iteration of the telehealth ambassador program.

### Conclusions

In summary, our first iteration of a stand-alone telehealth ambassador program to enhance patients’ digital literacy empowered a small group of patients to participate in video visits. As has been recommended by experts in the field, a multistakeholder and multipronged approach will be required to overcome inequities in telehealth video access [21]. The insights that we gleaned to enhance the reach and efficacy of the telehealth ambassador program must be considered alongside the limitations of our initial evaluation. Our small sample size limits the generalizability of our conclusions. Also, we did not collect patient-level data, so we were unable to discern whether individual sociodemographic characteristics were associated with program engagement and efficacy. Nevertheless, the data suggest that the program is valuable to a certain group of patients. A more robust version of the telehealth ambassador program will play a key role in our future approach to increasing the percentage of telehealth visits that are video versus audio-only. Increasing the reach of our existing telehealth ambassador program will require creative solutions that enhance widespread awareness of the benefits of video visits compared to audio-only visits and outreach solutions that do not rely solely on person power. Increasing the efficacy of our program will require multilevel interventions, including integration of the telehealth ambassador program into clinical workflows with routine and consistent recommendations for the use of telehealth video over audio-only visits, offering in-real-time IT support, and partnering with community-based organizations to enhance patient access to broadband and internet-enabled devices [22,23].

## Abbreviations

SFHN: San Francisco Health Network
